# Clinical and Genetic Factors Associated with Non-Response to Erenumab

**DOI:** 10.3390/jcm14248922

**Published:** 2025-12-17

**Authors:** Giulia Mallucci, Salvatore Terrazzino, Martina Giacon, Alberto Cordella, Sarah Cargnin, Christoph Schankin, Claudio Gobbi, Chiara Zecca

**Affiliations:** 1 Department of Neurology, Neurocenter of Southern Switzerland, Regional Hospital of Lugano, Ente Ospedaliero Cantonale, 6900 Lugano, Switzerland; 2 Department of Pharmaceutical Sciences, Università del Piemonte Orientale, 28100 Novara, Italy; 3 Department of Health Sciences, Università del Piemonte Orientale, 28100 Novara, Italy; 4 Department of Neurology, Inselspital, Bern University Hospital, University of Bern, 3012 Bern, Switzerland; 5 Faculty of Biomedical Sciences, Università della Svizzera Italiana, 6900 Lugano, Switzerland

**Keywords:** anti CGRP antibodies, erenumab, migraine, treatment non-response

## Abstract

**Background**: Monoclonal antibodies targeting the calcitonin gene-related peptide (CGRP) pathway, such as erenumab (ERE), are effective migraine-preventive therapies for many patients. Identifying clinical and genetic factors associated with treatment failure is crucial for optimizing patient management. **Methods**: This multicenter, prospective observational study included patients with episodic or chronic migraine treated with ERE for 12 months. Demographics, migraine history, comorbidities, treatment outcomes, and genetic variants in CGRP receptor-related genes (CALCRL and RAMP1) were evaluated for associations with non-response to ERE, defined as a <50% reduction in monthly migraine days. **Results**: Of the 140 patients starting ERE, 11 were lost to follow up, 12 stopped ERE due to side effects; 18 patients were non-responders and were compared to 99 responders. Arterial hypertension [adjusted OR (aOR): 7.77, *p* = 0.007], smoking (aOR: 4.98, *p* = 0.014), and insomnia requiring medication (aOR: 4.51, *p* = 0.027) were associated with non-responder status. Genetic analysis revealed a nominal association between the RAMP1 rs6431564 polymorphism and non-responder status (nominal *p* = 0.025), which did not survive Bonferroni correction. The G allele was linked to a reduced risk (aOR per G allele: 0.28, *p* = 0.025) and caused the increased expression of RAMP1 in an allele-dose manner. **Conclusions**: Hypertension, smoking, insomnia requiring medication, and, nominally, the RAMP1 rs6431564 polymorphism were associated with non-responder status to ERE in migraine patients. Further validation of the present results in larger cohorts is needed.

## 1. Introduction

Monoclonal antibodies targeting the calcitonin gene-related peptide (CGRP) pathway, such as erenumab (ERE), represent a paradigm shift in the preventive treatment of migraine. For decades, migraine prophylaxis relied on repurposed medications from other therapeutic classes, often with limited efficacy and poorly tolerated side effects [1,2]. The development of targeted CGRP pathway therapies marked the beginning of a new era in migraine-specific pharmacology.

ERE received approval based on randomized controlled trials demonstrating significant reductions in monthly migraine days and improved responder rates compared with placebo in both episodic [3,4] and chronic migraine populations [5]. These trials established its efficacy and safety profile.

However, research and clinical practice have shown that the effectiveness of these treatments varies, with some patients deriving little to no benefit from these therapies [3,4,5,6,7,8]. This variability poses a major clinical challenge, as a number of patients discontinue treatment due to a perceived lack of efficacy, leading to patient frustration and the inefficient use of costly healthcare resources.

Treatment response to ERE may be influenced by demographic and clinical features of the patient, but may also depend on their genetic profile. Specifically, the CGRP receptor targeted by ERE is a heterodimer formed by the calcitonin receptor-like (CALCRL) gene product and the receptor-activity-modifying protein 1 (RAMP1). As genetic variation in pharmacodynamic targets can influence treatment outcomes [9], polymorphisms in CALCRL and RAMP1 may alter the efficacy of ERE by changing the structure, expression, or binding affinity of its receptor complex. This pharmacogenetic approach, although still in its infancy for anti-CGRP therapies [10,11], holds great potential for personalizing treatment by uncovering the biological basis of this variable response and ultimately enabling the development of predictive biomarkers.

A primary goal of pharmacogenetic research in this area is therefore not only to predict success, but also to anticipate failure. Indeed, recognition of patients unlikely to respond to ERE using clinical and genetic factors can help avoid ineffective treatments, facilitating quicker transitions to alternative therapies and ultimately reducing patients’ burdens and healthcare costs.

To address this gap, this study investigated clinical, demographic, and genetic factors associated with non-responder status to ERE treatment in patients with episodic or chronic migraine, with a particular focus on the role of common polymorphisms in the CALCRL and RAMP1 genes, as potential predictors of treatment failure in a real-world tertiary care setting in Switzerland. We hypothesized that common polymorphisms within CALCRL and RAMP1 could be significantly associated with a higher likelihood of ERE discontinuation due to perceived inefficacy.

## 2. Methods

This investigation included patients who were already part of a previous study reported elsewhere (*n* = 110) [11], and an additional 30 patients were included at a later stage. The study was a Swiss multicenter, observational, prospective, and exploratory investigation that involved episodic or chronic migraine patients aged 18 to 70 years.

Participants had to fulfill the Swiss reimbursement criteria for ERE, that is, to experience at least 8 monthly migraine days (MMDs) documented for at least 3 months preceding treatment and to have experienced failure, intolerability, or contraindications to at least two previous migraine-preventive therapies approved in Switzerland (beta blockers, antidepressants, calcium antagonists, or anticonvulsants). The main exclusion criteria were botulinum toxin injections within 4 months prior to inclusion; initiation or dose change of a migraine-preventive medication within 2 months prior to inclusion; presence of primary or secondary headaches other than migraine; or contraindications to ERE.

Included patients initiated ERE 70 mg according to their neurologists’ clinical judgment between December 2019 and January 2023 and were followed according to clinical practice. Data were collected 3 months before the first ERE 70 mg injection, at the time of the first ERE 70 mg injection (baseline), and 3 and 12 months after treatment start, while continuing to maintain a headache diary. According to Swiss label, in case of reduction in monthly migraine days (MMDs) of at least 30% but < 50% 3 months after treatment start, ERE dose could be increased to 140 mg per month.

**Figure 1:**
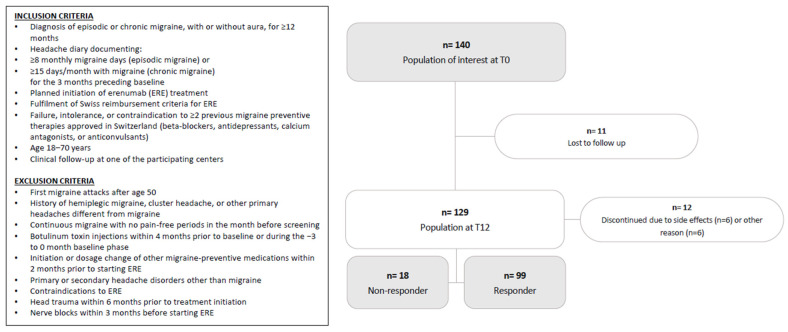
Flow chart of study population.

In total, 140 initiated ERE, of whom 129 completed the 12-month follow-up. (Figure 1)

Baseline data included socio-demographic characteristics (age, sex, BMI, menopause in women y/n, previous pregnancy y/n, working status employed/unemployed/retired, smoking status y/n, alcohol intake y/n, physical activity y/n, civil status single/married/other, insomnia absent/present, insomnia w/o medication, snoring y/n, anxiety y/n, depression y/n, chronic pain y/n, hypertension y/n, other comorbidities y/n, head trauma y/n, and number of first degree relatives with migraine), migraine history (migraine form episodic/chronic, age at migration onset, and number of failed preventive treatments), and genetic analysis of single nucleotide polymorphisms (SNPs) at CALCRL and RAMP1 genes, which encode subunits of the CGRP receptor. The data collected at baseline and during the 3 months before and 3 and 12 months after starting ERE included the number of MMDs, the monthly number of days with triptan/non-steroidal analgesic use, average pain intensity (visual analog score, VAS), attack duration, presence of medication overuse y/n, and adverse events. Additionally, HIT (Headache Impact Test)-6 score and MIDAS (Migraine Disability Assessment Score) score were collected. Briefly, HIT-6 scores range from 36 to 78, with scores ≥ 60 indicating severe impact. MIDAS scores categorize disability as follows: 0–5 (little or no disability), 6–10 (mild), 11–20 (moderate), and ≥21 (severe disability).

Patients were categorized into 2 groups:

1. Patients who discontinued ERE due to insufficient treatment response, defined as a <50% reduction in MMDs, according to the Swiss reimbursement requirement (non-responders, NRESP).
2. Patients who had an MMDs reduction ≥50% at 12 months (“responders”, RESP).

The aim of the study was to assess demographic, clinical, and genetic features associated with NRESP vs. RESP status.

### 2.1. Pharmacogenetic Analysis

Genomic DNA was extracted from whole blood samples using the QiaAmp DNA Mini Kit (Qiagen, Milan, Italy). Genotyping of CALCRL and RAMP1 polymorphisms was performed by real-time PCR using Applied Biosystems TaqMan Pre-Designed SNP Genotyping assays (CALCRL rs6710852 assay ID: C_189160430_10; RAMP1 rs13386048 assay ID: C__31241845_10; RAMP1 rs12465864 assay ID: C__11739774_10; RAMP1 rs6431564assay ID: C___2149740_10). These 4 SNPs were selected based on our previous publication in which they were associated with positive clinical improvement at month 3 after ERE start [12]. Our rationale was that a genetic variant capable of influencing treatment success is also the most logical candidate for investigating treatment failure, as both outcomes are likely due to the same underlying pharmacogenetic mechanisms. Genotyping was performed in accordance with previously validated protocols [13].

### 2.2. Statistical Analysis

Categorical data have been summarized in counts and percentages; continuous data have been described by mean and standard deviation when distributed normally. In case of deviation from normality, median and interquartile range (IQR) were used. To compare differences in clinical variables between the two patient groups (RESP vs. NRESP), the Student *t*-test was applied for continuous variables with equal variances and the nonparametric Mann–Whitney test for those with unequal variances, while the chi-squared test was used for assessing differences in the distribution of categorical variables. Deviation from Hardy–Weinberg equilibrium (HWE) was assessed for each polymorphism using a Chi-square goodness-of-fit test with 1 degree of freedom, using an online calculator (https://www.sebc.me/bioblog/labs/hwe-calculator) (accessed on 2 September 2025). Deviation from HWE was defined as a *p*-value < 0.05. For the comparison of continuous variables in the same subject, at baseline and up to 12 months after treatment start, the Wilcoxon test for paired samples was used. The association of SNPs with NRESP status was assessed by crude (i.e., unadjusted) and adjusted logistic regression analysis by confounding clinical variables (cut-off of *p*-value < 0.1 from univariate analyses). The crude and adjusted odds ratios (ORs) and the associated 95% confidence intervals (CIs) were calculated assuming an additive genetic model of inheritance, where the reported OR represents the increase in risk for each individual copy of the variant allele. The major allele was used as the reference, and the minor allele was considered the effect allele for all analyses. Finally, variables with a *p*-value < 0.05 from the univariate analyses were included in a multivariate logistic regression model to identify independent factors associated with NRESP status. Missing data were not imputed; therefore, a complete case analysis was performed for the multivariate logistic regression. The data were managed with the software MedCalc version 13.3.3 (MedCalc Software; Mariakerke, Belgium). Given the exploratory nature of our pharmacogenetic study, we considered nominal *p* values based on two-sided tests with a = 0.05. However, to reduce the risk of chance results due to multiple testing, the adjusted *p*-value based on Bonferroni correction was also considered, and the threshold of statistical significance was lowered to *p* < 0.0125 (i.e., 0.05/4) to account for a total of 4 polymorphisms analyzed. Retrospective power calculations were performed using Quanto software (version 1.2.4, University of Southern California, CA USA) [14] to evaluate the statistical power of the final sample size. The correlation of SNPs with gene expression was assessed using the Genotype-Tissue Expression (GTEx) (Version: V10, https://www.gtexportal.org/home) data [15].

### 2.3. Standard Protocol Approvals, Registrations, and Patient Consents

The study adhered to the World Medical Association’s Declaration of Helsinki and received approval from the local ethics committees of the participating centers (the Ethical Cantonal Committee of Bellinzona, (approval number: 2019-01393 CE 3507, approval date: 10 September 2019). Written informed consent for the use of clinical data was obtained from all participants.

### 2.4. Data Availability

Individual de-identified participant data will be shared on reasonable request by professionals in this field.

## 3. Results

### 3.1. Study Population

In total, 140 patients initiated ERE, of whom 129 completed the 12-month follow-up.

During the study, 11 patients were lost to follow-up. Of the remaining 129 participants, 58 switched from ERE 70 mg to ERE 140 mg. The median time to switch to ERE 140 was 169.5 days (IQR 115.0–222.0).

Twelve patients discontinued ERE between months 3 and 12 due to side effects (*n* = 5 on ERE 70 mg; *n* = 1 on ERE 140 mg) or for reasons unrelated to lack of effectiveness: *n* = 1 died due to complications from melanoma (ERE 70 mg); *n* = 2 discontinued because their health insurance refused further reimbursement (ERE 70 mg); *n* = 2 stopped for personal reasons (ERE 140 mg); and *n* = 1 discontinued due to plans for pregnancy (ERE 70 mg).

Ninety-nine patients (median (IQR) age 49.6 (37.2–55.0) years, 87.9% females) completed the 12-month treatment course and had a reduction in MMDs of ≥50% at month 12, being therefore classified as RESP. Eighteen patients (median [IQR] age 49.3 (46.3–53.3) years, 66.7% females) were classified as NRESP, as they discontinued treatment due to a <50% reduction in MMDs, after a median (IQR) treatment duration of 3.5 (1–6) months (nine patients discontinued ERE within the first 3 months of treatment, and nine between months 4 and 12).

The complete characteristics of the study participants are summarized in Table 1 and Table 2.

### 3.2. Clinical Course During Study Period

Compared to baseline values at their last follow-up, NRESP had an unchanged median (IQR) number of MMDs of 11.0 (9.7–30.0) vs. 12.0 (7.5–29.2), *p* = 0.765. The median (IQR) pain intensity (VAS), duration of attacks, and triptan use did not show significant changes [8.0 (7.0–8.0) vs. 7.0 (5.7–8.0), *p* = 0.063; 6.0 (2.9–24.0) vs. 4.0 (2.0–24.0) hours, *p* = 0.156; 0 (0–8.0) vs. 0 (0–8.2), *p* = 0.195, respectively]. The use of non-triptan analgesics decreased from a median of 6.0 (1.5–22.5) to 4.0 (0–17.5) days (*p* = 0.016). The median (IQR) HIT-6 score decreased from 67.0 (63.7–70.5) to 63.0 (59.5–68.5, *p* = 0.032), though the median (IQR) MIDAS score did not change significantly (40.0 (15.0–67.5) to 30.0 (15.0–64.5), *p* = 0.588).

As for RESP, the median (IQR) MMDs decreased from 15.5 (11.0–24.0) to 4.0 (2.3–7.3; *p* < 0.0001). Consistently, marked reductions were seen in the frequency of use of triptans and non-triptan analgesics, pain intensity and duration, and migraine-related burden as measured with HIT-6 and MIDAS (*p* < 0.0001 for all comparisons; see Supplementary Tables S1 and S2).

### 3.3. Clinical Factors Associated with NRESP Status

The univariate analysis revealed several differences between RESP and NRESP (Table 1 and Table 2).

Male sex was associated with NRESP status (33.3% vs. 12.1% of RESP, *p* = 0.022). Current or past smoking status also showed association with NRESP status (72.2% vs. 39.6% of RESP, *p* = 0.001), as did arterial hypertension (38.9% vs. 8.2% of RESP, *p* < 0.001) and insomnia treated with medication (55.6% vs. 32.0% of RESP, *p* = 0.005).

There were some trends for an association with NRESP status observed for higher BMI, use of triptans on fewer days at baseline, and lower pain intensity at baseline.

Other variables, including age, age at migraine onset, number of failed preventive medications, HIT-6 and MIDAS scores at baseline menopausal status, pregnancy history, working status, family history of migraine, alcohol intake, physical activity, anxiety, depression, chronic pain, migraine form, and the use of concomitant preventive medications, were not associated with NRESP status.

All variables showing a significant association in the univariate analysis (*p* < 0.05), namely sex, smoking status, insomnia treated by medication, and arterial hypertension, were included in the subsequent multivariate logistic regression model. In the multivariate analysis (Table 3), smoking status (OR 4.98, 95% CI 1.38–17.93, *p* = 0.014), insomnia treated with medication (OR 4.51, 95% CI 1.19–17.14, *p* = 0.027) and arterial hypertension (OR 7.77, 95% CI 1.76–34.30, *p* = 0.007) remained significantly associated with NRESP status, while sex did not (OR 2.38, 95% CI 0.59–9.70, *p* = 0.230).

### 3.4. Genetic Factors Associated with NRESP Status

Genotype distributions for all analyzed polymorphisms were consistent with the Hardy–Weinberg equilibrium (CALCRL rs6710852, *p* = 0.886; RAMP1 rs13386048, *p* = 0.553; RAMP1 rs12465864, *p* = 0.699; RAMP1 rs6431564, *p* = 0.120). Neither in the univariate logistic regression analysis nor after adjustment for the potential confounders—male sex, insomnia treated with medication, hypertension, and smoking status—did any of the four analyzed polymorphisms (CALCRL rs6710852, RAMP1 rs13386048, RAMP1 rs12465864, and RAMP1 rs6431564) reach statistical significance when applying Bonferroni correction for multiple testing (Bonferroni *p*-value threshold <0.0125). Nonetheless, after adjusting for clinical confounders, a nominal association with NRESP status was found for the RAMP1 rs6431564 locus, which had an adjusted OR of 0.28 (95% CI 0.09–0.86, *p* = 0.025) in the additive genetic model, indicating a lower risk of treatment inefficacy for each G allele of RAMP1 rs6431564. The other polymorphisms analyzed, including CALCRL rs6710852, RAMP1 rs13386048, and RAMP1 rs12465864, showed no nominal association after adjustment, with *p*-values of 0.739, 0.820, and 0.664, respectively (Table 4).

### 3.5. Correlation of RAMP1 rs6431564 with Gene Expression

**Figure 2:**
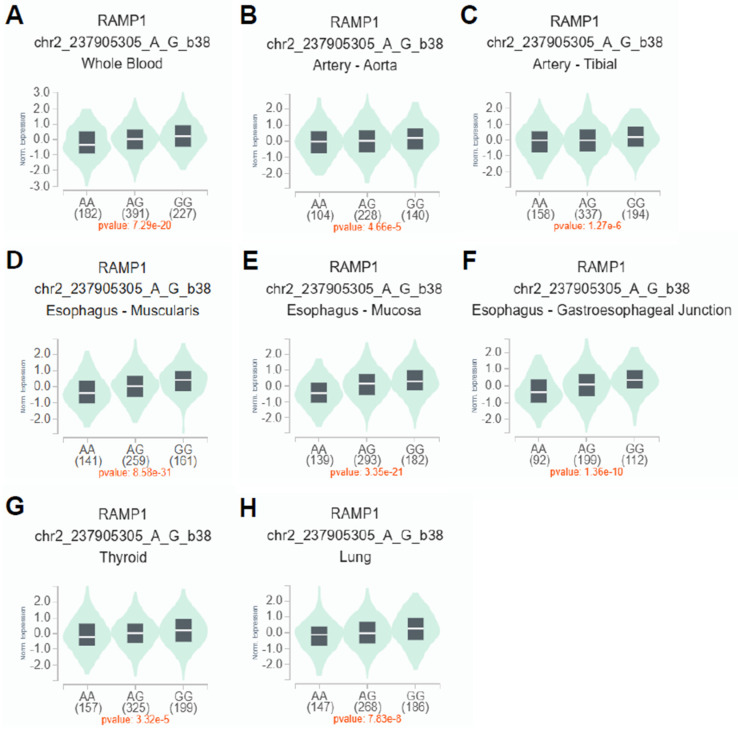
Expression quantitative trait loci (eQTL) analysis of RAMP1 rs6431564 A>G polymorphism in different tissues (all *p* < 0.001) using the GTEx portal database (https://www.gtexportal.org/home/). The plots display the normalized expression of RAMP1 mRNA by genotype (AA, AG, GG) in the following tissues: (**A**) Whole Blood; (**B**) Artery–Aorta; (**C**) Artery–Tibial; (**D**) Esophagus–Muscularis; (**E**) Esophagus–Mucosa; (**F**) Esophagus–Gastroesophageal Junction; (**G**) Thyroid; and (**H**) Lung. Note: The *p*-values and axis labels in this figure are presented in the standard output format of the GTEx software, where “e” indicates exponential scientific notation (e.g., 7.29e-20 represents 7.29 × 10^−20^) and hyphens rep-resent negative values.

To investigate the impact of rs6431564 A>G on RAMP1 mRNA expression, cis-expression quantitative trait loci (eQTL) analysis was performed using GTEx in Single-Tissue eQTLs analysis (Figure 2). The results showed that rs6431564 A>G is significantly related to an allele-dose-dependent increase (with additive effect of G allele) of RAMP1 mRNA expression in different tissues: whole blood (*p* = 7.29 × 10^−20^, Figure 2A), artery–aorta (*p* = 4.66 × 10^−5^, Figure 2B), artery–tibial (*p* = 1.27 × 10^−6^, Figure 2C), esophagus–muscularis (*p* = 8.58 × 10^−31^, Figure 2D), esophagus–mucosa (*p* = 3.35 × 10^−21^, Figure 2E), esophagus–gastroesophageal Junction (*p* = 1.36 × 10^−10^, Figure 2F), thyroid (*p* = 3.32 × 10^−5^, Figure 2G), and lung (*p* = 7.83 × 10^−8^, Figure 2H).

## 4. Discussion

While much of the literature has focused on identifying predictors of a positive response to CGRP-targeted treatments [16], our study highlights key factors associated with non-response to ERE, including hypertension, smoking, insomnia treated with medication, and RAMP1 rs6431564 polymorphism. Recognizing these clinical and genetic predictors of non-response to ERE is crucial to guide the selection of patients who will likely profit most from this treatment, thus avoiding useless exposure to an ineffective therapy, improving migraine management, and ultimately optimizing healthcare resources.

A significant finding was that patients who required medication for insomnia were more likely to be classified as NRESP. Insomnia and migraine have a well-documented bidirectional relationship, where poor sleep can both trigger migraines and exacerbate their severity, while chronic migraines disrupt sleep patterns [17]. Research has shown that patients with co-existing insomnia tend to experience more frequent and severe migraines, which can, in turn, reduce the effectiveness of preventive migraine treatments [18]. There is also emerging evidence that anti-CGRP therapy could influence sleep patterns in migraine patients by modulating pathways involved in circadian regulation [19]. The relationship between the CGRP pathway and sleep or circadian rhythm might be modulated by melatonin, which has been shown to reduce CGRP release in different in vivo and in vitro models [20]. Additionally, one study in Drosophila models demonstrated that the “loss of function” of a homolog of the CGRP receptor resulted in better sleep, particularly in the second half of the night [21]. Importantly, melatonin has been studied in migraine prophylaxis with positive results [17].

We also observed a significant association between smoking and an increased risk of being a NRESP to ERE. Some studies suggest that smoking is more prevalent in individuals with migraines, especially those with more severe symptoms and medication overuse [22], suggesting that smoking might negatively impact migraine severity and the likelihood of treatment response. The exact nature of this relationship remains, however, speculative.

We found a strong association between arterial hypertension and an increased likelihood of being a NRESP to ERE, consistent with our previous findings that linked arterial hypertension to a reduction of more than 90% in the probability of treatment efficacy [12]. Of note, the population of this present study has been enriched by almost 30% with additional patients and reinforcing our previous result. One possible explanation is that ERE is known to potentially increase arterial blood pressure; therefore, we cannot completely exclude a role of insufficiently controlled arterial hypertension in the genesis of headache in NRESP patients. This effect might be even more pronounced in hypertensive patients with migraine due to compromised vascular function, such as increased arterial stiffness or endothelial dysfunction [23]. Our identification of comorbidities as key predictors of treatment failure aligns with findings from recent large-scale, real-world studies and meta-analyses [16,24], which consistently suggest that a higher burden of concomitant diseases and more refractory migraine phenotypes are associated with lower response rates to anti-CGRP monoclonal antibodies.

Notably, factors such as higher BMI, male sex, and depression, which were associated with a higher likelihood of being NRESP to ERE in the univariate analysis, did not survive in the multivariate model. This suggests that these variables may be confounded by other factors or that their impact is of a lesser magnitude and cannot be detected within our study population, probably due to the small sample size. Of particular note, our study confirms that there is no difference in the efficacy of CGRP-targeted therapies for migraine prevention between males and females. Although earlier clinical trials suggested reduced efficacy in males, these trials were likely underpowered for such a subgroup analysis. In addition, more recent real-world evidence, along with our findings, indicates that CGRP-targeting therapies demonstrate similar efficacy across both sexes, particularly in chronic migraine cases [25]. Given the limited sample size, a more in-depth sex-based analysis was not feasible. Future larger studies are needed to clarify any subtle sex-related differences in treatment response.

Regarding genetic factors, our analysis revealed no association between the studied genetic polymorphisms and ERE NRESP status when the conservative Bonferroni method was used to correct for multiple testing. However, the additive model for the RAMP1 rs6431564 polymorphism showed a nominal association with NRESP status after adjusting for confounding factors, with the G allele being associated with a lower probability of treatment inefficacy. The fact that this association emerged only after adjustment suggests the presence of negative confounding, where the strong influence of clinical risk factors such as hypertension likely masked the underlying genetic effect in the unadjusted analysis. By statistically controlling for these confounding factors, the model was able to unmask the independent contribution of genotype. Although this result did not survive the stringent Bonferroni correction, we believe it is worth reporting due to the exploratory nature of our study and the strong biological plausibility of a variant in the drug’s target receptor gene affecting its efficacy. It is noteworthy that GTEx analysis revealed an effect of rs6431564 on RAMP1 mRNA expression in different tissues, with the G allele causing increased expression in an allele-dose manner. This finding and the pharmacogenetic results suggest that genetic predisposition to increased expression of RAMP1 correlates with a lower likelihood of being NRESP to ERE. Since RAMP1 heterodimerizes with CALCRL to form the canonical CGRP receptor—the direct target of ERE—increased transcription of RAMP1 is expected to expand the pool of functional receptor complexes on the cell surface. Our data suggest that increased RAMP1 expression mediated by the G allele of rs6431564 favors more efficient antibody binding or promotes receptor internalization after ligation, ultimately enhancing pharmacodynamic blockade of the CGRP pathway. Nevertheless, alternative explanations—such as linkage to nearby functional variants that influence post-translational modification or transport of the receptor—cannot be ruled out. However, further investigations in larger pharmacogenetic studies are needed to confirm the clinical relevance of this polymorphism and to explore other genetic markers that may predict treatment outcome.

There is currently very little information on the influence of genetic variants on the response of migraine patients to anti-CGRP monoclonal antibodies. Recently, in a retrospective cohort of 199 migraine patients treated with different anti-CGRP monoclonal antibodies, of which 51.7% of patients were treated with ERE, a nominal association was found between RAMP1 rs12615320 and non-responder status as assessed by the MIDAS question A [26]. Because we did not genotype rs12615320, we assessed its linkage disequilibrium (LD) with rs6431564 using *LDpair* and *LDhap* [27]. We found that these two SNPs are in LD (D’ = 0.53, R2 = 0.01, *p* = 0.0007). Despite the differences in study design, criteria for defining non-responders, and timing of assessment of responder status, the observation in two independent studies that two SNPs of RAMP1 in LD are both nominally associated with non-response to anti-CGRP monoclonal antibodies may suggest LD of these two SNPs with an unidentified SNP more strongly associated with risk of non-response to ERE efficacy. Fine mapping of the RAMP1 gene region containing rs6431564 and rs12615320 and subsequent functional SNP analysis are required to test this hypothesis and find the true causal SNP for non-response to ERE.

On the other hand, six SNPs found in a prospective, observational genome-wide association study (GWAS) in a cohort of 108 Han Chinese patients with CM who had received fremanezumab or galcanezumab for at least 12 weeks, associated with treatment response with a threshold of *p* < 1 × 10^−7^ (rs116870564 in LRRC4C, rs75244870 near ACOX2, rs56216870 near MTSS1, rs12938101 near TMEM92-AS1, rs74655790 in ATAD2B and rs149540851 in OXR1) [28]. Among these six SNPs, the strongest association was found with the intronic variant rs116870564 in the LRRC4C gene (*p* = 6.65 × 10^−9^), which is known to play a role in axon guidance and synaptic plasticity [29]. How LRRC4C rs116870564 might be related to the pharmacodynamics of the anti-CGRP monoclonal antibodies fremanezumab and galcanezumab remains to be determined. In contrast to the hypothesis-free, short-term GWAS described above, our pharmacogenetic study relied on a follow-up period of 1 year to evaluate the efficacy of ERE. The potential differences between the short- and long-term outcomes of patients receiving anti-CGRP monoclonal antibody therapy have been brought to light by recent studies. For example, a multicenter study showed little agreement between response rates at 3 and 12 months, suggesting that early response may not be a reliable predictor of long-term treatment efficacy of CGRP monoclonal antibodies [30]. In addition, our pharmacogenetic study has a strong rationale, as it was based on polymorphic variants in genes that are plausible candidates for the pharmacodynamics of ERE and thus for the clinical response to the drug.

Our study has some limitations. First, it was a post hoc analysis with a retrospective design and a relatively small sample size, especially in the NRESP group. This critical limitation reduces the statistical power to detect associations with small to moderate effect sizes, increases the risk of false-negative results, and makes all identified associations less robust. Given the sample of 99 RESP and 18 NRESP, a retrospective power calculation confirmed that the study did not have sufficient statistical power to detect modest odds ratios for the SNPs studied. This power limitation is clear when examining the minimum effect sizes required for detection. To achieve 80% power at a nominal significance level of 0.05, the minimum detectable ORs were 0.256 (inverse 3.9) for CALCRL rs6710852 (MAF: 0.11), 0.294 (inverse 3.4) for RAMP1 rs12465864 (MAF: 0.16), 0.345 (inverse 2.9) for RAMP1 rs13386048 (MAF: 0.33), and 0.344 (inverse 2.9) for RAMP1 rs6431564 (MAF: 0.48). Second, our genetic results should be interpreted with caution. The nominal association of RAMP1 rs6431564 with ERE NRESP status did not survive conservative Bonferroni correction for multiple testing. Although we argue for its relevance based on biological plausibility, this lack of statistical robustness means that the result should be considered exploratory and hypothesis-generating until replication. Because of our small sample size, we were also not able to conduct a separate analysis on factors associated with ERE discontinuation due to tolerability issues [24,31]. Third, the observational design precludes establishing definite causal relationships. Despite adjustment for relevant clinical covariates, the influence of unmeasured confounders—such as detailed psychological profiles, specific lifestyle changes, dietary habits, or precise adherence patterns beyond diary reporting—cannot be ruled out. Regarding generalizability, recruitment from a specialized tertiary care center under strict Swiss reimbursement criteria (requiring failure of at least two prior prophylactic classes) introduces a selection bias toward patients with a more refractory migraine phenotype. Therefore, our findings may not fully apply to the general migraine population or to healthcare settings with less restrictive access to anti-CGRP therapies. Additionally, while the 12-month follow-up provides a robust assessment of mid-term effectiveness, it limits our ability to evaluate the long-term duration of the clinical response or the possibility of secondary loss of effectiveness over an extended treatment period. Finally, we lacked an independent patient cohort to validate our results. External validation is critical for confirming the reliability of clinical and genetic predictors, and its absence limits the strength of our conclusions. Replication in larger cohorts and dedicated functional studies (e.g., quantification of CALCRL–RAMP1 complexes at the surface and ERE binding kinetics in genotype-stratified cell models) are required to validate RAMP1 rs6431564 as a predictive biomarker. If confirmed, the genotyping of this single SNP could represent a pragmatic step towards personalized anti-CGRP therapy, allowing physicians to anticipate the effectiveness of ERE and refine treatment selection at an early stage. To build on our findings, future research should also perform a comprehensive sequencing of the CGRP pathway genes. This approach is critical for identifying a broader range of genetic variants that, in conjunction with clinical data, can improve the development of robust predictive algorithms and ultimately inform treatment decisions.

## 5. Conclusions

This study highlights key clinical and genetic factors associated with non-response to ERE in migraine patients. Clinical factors included insomnia requiring medication, smoking, and arterial hypertension. The RAMP1 rs6431564 polymorphism showed a potential genetic association with NRESP status, though further validation is needed.

## Figures and Tables

**Table 1:** Univariate association analysis of clinical continuous variables.

Clinical Variable	RESP (*n* = 99) Median (IQR)	NRESP (*n* = 18) Median (IQR)	*p*-Value
**Age, years**	49.6 (37.2–55.0)	49.3 (46.3–53.3)	0.806
**Age at migraine onset (missing, *n* = 1), years**	15.0 (12.0–20.0)	15.0 (13.0–23.0)	0.488
**BMI (missing, *n* = 2), mean (SD), kg/m^2^**	23.1 (3.7)	24.9 (4.8)	0.076
**Number of failed preventive medications**	3 (2.0–4.0)	3.5 (2.0–6.0)	0.311
**Number of first-degree relatives with migraine (missing, *n* = 1)**	1 (0–2.0)	1 (0–1.0)	0.458
**Average attack duration at baseline (missing, *n* = 2), hours**	18.0 (4.0–31.5)	7.0 (3.0–24.0)	0.317
**Average pain intensity at baseline, VAS**	8.0 (8.0–9.4)	8.0 (7.0–8.0)	0.078
**HIT-6 score at baseline (missing, *n* = 1)**	68.0 (64.0–70.0)	66.5 (63.0–70.0)	0.479
**MIDAS at baseline, score**	45.5 (26.0–90.0)	38.5 (15.0–65.0)	0.233
**Monthly days with triptan use at baseline, days**	6.0 (0–11.25)	0 (0–8.0)	0.075
**Monthly days with use of non-triptan analgesics at baseline (missing, *n* = 1), days**	5.5 (0–13.0)	5.5 (0–20.0)	0.466

HIT-6, Headache Impact Test-6; IQR, interquartile range; MIDAS: Migraine Disability Assessment Score, RESP: responders; NRESP: non-responders; VAS: visual analog scale.

**Table 2:** Univariate association analysis of clinical categorical variables.

Clinical Variable	RESP (*n* = 99) *n* (%)	NRESP (*n* = 18) *n* (%)	*p*-Value
**Sex, *n* (%)**			**0.022**
Female Male	87 (87.9) 12 (12.1)	12 (66.7) 6 (33.3)	
**Migraine form**			0.432
Episodic Chronic	45 (45.5) 54 (54.5)	10 (55.6) 8 (44.4)	
**Concomitant preventive medications (missing, *n* = 1)**			0.426
No Yes	31 (31.6) 67 (68.4)	4 (22.2) 14 (77.8)	
**Use of triptans**			0.086
No Yes	29 (29.3) 70 (70.7)	9 (50.0) 9 (50.0)	
**Switch to ERE 140 mg**			0.638
No Yes	49 (49.5) 50 (50.5)	10 (55.6) 8 (44.5)	
**Insomnia (missing, *n* = 2)**			**0.005**
Absent Present + medication Present − medication	47 (48.5) 19 (19.6) 31 (32.0)	5 (27.8) 10 (55.6) 3 (16.7)	
**Hypertension (missing, *n* = 2)**			**<0.001**
Absent Present	89 (91.8) 8 (8.2)	11 (61.1) 7 (38.9)	
**Menopause in women (*n* = 99)**			0.817
Absent Present	55 (63.2) 32 (36.8)	8 (66.7) 4 (33.3)	
**Pregnancy (*n* = 99)**			0.780
No Yes	40 (46.0) 47 (54.0)	5 (41.7) 7 (58.3)	
**Working status (missing, *n* = 2)**			0.334
Employed Unemployed Retired	65 (67.0) 25 (25.8) 7 (7.2)	9 (50.0) 8 (44.4) 1 (5.6)	
**First-degree relatives with migraine (missing, *n* = 1)**			0.877
No Yes	29 (29.6) 69 (70.4)	5 (27.8) 13 (72.2)	
**Smoking status (missing, *n* = 2)**			**0.001**
Never Current or past	59 (60.4) 38 (39.6)	5 (27.8) 13 (72.2)	
**Alcohol intake (missing, *n* = 3)**			0.845
No Yes	61 (63.5) 35 (36.5)	11 (61.1) 7 (38.9)	
**Physical activity (missing, *n* = 3)**			0.389
Absent Present	48 (50.0) 48 (50.0)	11 (61.1) 7 (38.9)	
**Civil status (missing, *n* = 2)**			0.387
Single Married Other	25 (25.8) 57 (58.8) 15 (15.5)	5 (27.8) 8 (44.4) 5 (27.8)	
**Snoring (missing, *n* = 2)**			0.624
Absent Present	70 (72.2) 27 (27.8)	14 (77.8) 4 (22.2)	
**Anxiety (missing, *n* = 1)**			0.610
Absent Present	50 (51.0) 48 (49.0)	8 (44.4) 10 (55.6)	
**Depression (missing, *n* = 1)**			0.146
Absent Present	51 (52.0) 47 (48.0)	6 (33.3) 12 (66.7)	
**Chronic pain (missing, *n* = 1)**			0.206
Absent Present	74 (75.5) 24 (24.5)	11 (61.1) 7 (38.9)	
**Other comorbidities (missing, *n* = 1)**			0.548
Absent Present	67 (68.4) 31 (31.6)	11 (61.1) 7 (38.9)	
**Head trauma (missing, *n* = 2)**			0.348
Absent Present	77 (79.4) 20 (20.6)	16 (88.9) 2 (11.1)	

*p*-values in bold are statistically significant (*p* < 0.05). RESP, responders.

**Table 3:** Multivariate association analysis of clinical factors predicting NRESP status.

Clinical Variable	OR (95%)	*p*-Value
**Sex**		
Female	1 (Ref)	
Male	2.38 (0.59–9.70)	0.230
**Smoking status**		
Never	1 (Ref)	
Current or past	4.98 (1.38–17.93)	**0.014**
**Insomnia**		
Absent	1 (Ref)	
Present + medication	4.51 (1.19–17.14)	**0.027**
Present − medication	0.54 (0.09–3.16)	0.498
**Hypertension**		
Absent	1 (Ref)	
Present	7.77 (1.76–34.30)	**0.007**

*p*-values in bold are statistically significant (*p* < 0.05). NRESP: non-responder, OR: odds ratio.

**Table 4:** Association analysis of SNPs with NRESP status.

SNP	All Patients (*n* = 140) *n* (%)	Resp (*n* = 99) *n* (%)	No Resp (*n* = 18) *n* (%)	Crude OR (95% CI)	*p*-Value	Adjusted OR * (95% CI)	*p*-Value
**CALCRL rs6710852**							
TT	110 (78.6)	75 (75.8)	15 (83.3)	0.61 (0.17–2.22)	0.456	0.61 (0.15–2.39)	0.473
TG	28 (20.0)	23 (23.2)	3 (16.7)				
GG	2 (1.4)	1 (1.0)	0 (0)				
**RAMP1 rs13386048**							
GG	64 (45.7)	50 (50.5)	7 (38.9)	1.38 (0.69–2.76)	0.368	1.30 (0.55–3.04)	0.550
GA	59 (42.1)	37 (37.4)	8 (44.4)				
AA	17 (12.1)	12 (12.1)	3 (16.7)				
**RAMP1 rs12465864**							
AA	98 (70.0)	69 (69.7)	14 (77.8)	0.84 (0.30–2.31)	0.732	1.21 (0.39–3.81)	0.742
AG	39 (27.9)	28 (28.3)	3 (16.7)				
GG	3 (2.1)	2 (2.0)	1 (5.6)				
**RAMP1 rs6431564**							
AA	34 (24.3)	24 (24.2)	5 (27.8)	0.65 (0.29–1.42)	0.278	0.28 (0.09–0.86)	**0.025**
AG	79 (56.4)	55 (55.6)	12 (67.7)				
GG	27 (19.3)	20 (20.2)	1 (5.6)				

Association analysis was performed using an additive genetic model. Crude OR refers to the unadjusted odds ratio (risk per copy of the minor allele), while Adjusted OR * is adjusted for gender, insomnia, hypertension, and smoking status. *p*-values in bold indicate statistical significance. CI: confidence interval; NRESP: non-responder; OR: odds ratio; RESP: responder.

## Data Availability

The data presented in this study are available on reasonable request from the corresponding author.

## Supplementary Materials

The following supporting information can be downloaded at: https://www.mdpi.com/article/10.3390/jcm14248922/s1, Table S1: Frequency, severity and impact of migraine as well as use of abortive therapies, before and at the last follow-up after ERE start, in the NRESP. Table S2:Frequency, severity and impact of migraine as well as use of abortive therapies before and during month 12 after ERE start in the RESP group.
