# Supplementary material for: Clinical and Genetic Factors Associated with Non-Response to Erenumab
Source: J Clin Med. 2025 Dec 17;14(24):8922. doi: 10.3390/jcm14248922 (PMC12733388; doi:10.3390/jcm14248922)
Supplement: Supplementary file 1 [file jcm-14-08922-s001.zip › jcm-4017393-supplementary.pdf]

Table Supplement 1: Frequency, severity and impact of migraine as well as use of abortive therapies, before and at the last follow-up after ERE start, in the NRESP group (n=18).

**Clinical variable**	**During the last 3 months before ERE start**	**At the last follow-up after ERE start**	**p-value**
MMD, median (IQR), n=17	11.0 (9.7-30.0)	12.0 (7.5-29.2)	0.765
Number of monthly days with triptan use, median (IQR), n=17	0 (0-7.2)	0 (0-8.2)	0.195
Number of monthly days with use of non-triptan analgesics, median (IQR), n=17	6.0 (1.5-22.5)	4.0 (0-17.5)	**0.016**
Pain intensity (VAS), median (IQR), n=17	8.0 (7.0-8.0)	7.0 (5.7-8.0)	0.063
Attack duration, hours, median (IQR), n=17	6.0 (2.9-24.0)	4.0 (2.0-24.0)	0.156
Hit-6 score, median (IQR), n=17	67.0 (63.7-70.5)	63.0 (59.5-68.5)	**0.032**
MIDAS score, median (IQR), n=17	40.0 (15.0-67.5)	30 (15.0-64.5)	0.588

ERE, erenumab; HIT-6, Headache Impact Test-6; IQR, interquartile range; MIDAS, Migraine Disability Assessment Score MMD; monthly migraine days; VAS, visual analog scale.

Table Supplement:2 Frequency, severity and impact of migraine as well as use of abortive therapies before and during month 12 after ERE start in the RESP group (n=99).

**Clinical variable**	**During the last 3 months before ERE start**	**At month 12 after ERE start**	**p-value**
MMD, median (IQR), n=74	15.5 (11.0-24.0)	4.0 (2.3-7.3)	<0.0001
Number of monthly days with triptan use, median (IQR), n=66	5.5 (0-12.0)	1.0 (0-3.3)	<0.0001
Number of monthly days with use of non-triptan analgesics, median (IQR), n=66	7.0 (0-13.0)	2.0 (0-4.7)	<0.0001
Pain intensity (VAS), median (IQR), n= 68	8.0 (8.0-10.0)	5.0 (4.0-7.0)	<0.0001
Attack duration, hours, median (IQR), n=67	13.0 (4.0-43.5)	3.0 (2.0-10.5)	<0.0001
HIT-6 score, median (IQR), n=65	68.0 (64.0-70.5)	52.0 (44.0-60.0)	<0.0001
MIDAS score, median (IQR), n=64	45.0 (27.0-81.0)	10.0 (1.0-23.0)	<0.0001

ERE, erenumab; HIT-6, Headache Impact Test-6; IQR, interquartile range; MIDAS, Migraine Disability Assessment Score MMD; monthly migraine days; VAS, visual analog scale.
